# Genomic Evolution of 11 Type Strains within Family Planctomycetaceae

**DOI:** 10.1371/journal.pone.0086752

**Published:** 2014-01-29

**Authors:** Min Guo, Qian Zhou, Yizhuang Zhou, Linfeng Yang, Tianxiang Liu, Jinlong Yang, Yanling Chen, Longxiang Su, Jin Xu, Jing Chen, Feng Liu, Jiapeng Chen, Wenkui Dai, Peixiang Ni, Chengxiang Fang, Ruifu Yang

**Affiliations:** 1 Shenzhen Key Laboratory of Environmental Microbial Genomics and Application, BGI-Shenzhen, Shenzhen, China; 2 Shenzhen Key Laboratory of Bioenergy, BGI-Shenzhen, Shenzhen, China; 3 BGI-Shenzhen, Shenzhen, China; 4 Medical College, Nankai University, Tianjin, China; 5 College of Life Sciences, Wuhan University, Wuhan, China; 6 State Key Laboratory of Pathogen and Biosecurity, Beijing Institute of Microbiology and Epidemiology, Beijing, China; Beijing Institute of Genomics, China

## Abstract

The species in family Planctomycetaceae are ideal groups for investigating the origin of eukaryotes. Their cells are divided by a lipidic intracytoplasmic membrane and they share a number of eukaryote-like molecular characteristics. However, their genomic structures, potential abilities, and evolutionary status are still unknown. In this study, we searched for common protein families and a core genome/pan genome based on 11 sequenced species in family Planctomycetaceae. Then, we constructed phylogenetic tree based on their 832 common protein families. We also annotated the 11 genomes using the Clusters of Orthologous Groups database. Moreover, we predicted and reconstructed their core/pan metabolic pathways using the KEGG (Kyoto Encyclopedia of Genes and Genomes) orthology system. Subsequently, we identified genomic islands (GIs) and structural variations (SVs) among the five complete genomes and we specifically investigated the integration of two Planctomycetaceae plasmids in all 11 genomes. The results indicate that Planctomycetaceae species share diverse genomic variations and unique genomic characteristics, as well as have huge potential for human applications.

## Introduction

The Planctomycetaceae family is distributed in both soil and water. Previous studies have estimated that 50% of the nitrogen molecules in the atmosphere are generated by anammox planctomycetes [1]. *Planctomyces bekefii* was the first member of family Planctomycetaceae, reported in 1924 by Gimesi, who originally established genus *Planctomyces* Gimesi 1924 to accommodate this peculiar aquatic “fungi” [2]. After nearly 50 years, Hirsch reported a freshwater bacterium in 1972 and named it “*Blastocaulis sphaerica*,” which was later identified as *P. bekefii* [3]. Further studies provided conclusive evidence that the two organisms are indistinguishable and both are bacteria rather than fungi [4]. The transmission electron micrographs of the negatively stained strains showed that Planctomycetaceae species possess crateriform structures on their cell surface. Some cells have stalks or extensive holdfast materials on one pole. To date, the Planctomycetaceae family includes 11 genera, namely, *Aquisphaera* [5], *Blastopirellula* [6], [7], *Gemmata* [8], *Isosphaera* [9], *Pirellula* [10], [11], *Planctomyces* [12], *Rhodopirellula* [6], *Schlesneria* [13], [14], *Singulisphaera* [14], [15], *Telmatocola* [16], and *Zavarzinella* [14], [17], with more than 18 species (http://www.bacterio.cict.fr/p/planctomycetaceae.html). The whole genomes of 11 type strains are available in the National Center for Biotechnology Information (NCBI) databases (ftp://ftp.ncbi.nlm.nih.gov/genomes/).

Planctomycetaceae species exhibit strong tolerance for seawater, acidic peat bogs, hot springs, and low temperatures [13], [15], [18]. Furthermore, members of Planctomycetaceae family possess unusual features, i.e., the absence of peptidoglycans, the synthesis of C_30_ sterols, the encoding of C_1_ transfer enzymes, and the presence of clathrin-like membrane coat proteins and anammoxosomes [18]. Their most unique feature is the presence of cell compartmentalization structure called a lipidic intracytoplasmic membrane (ICM) [18], which is unusual in prokaryotic and eukaryotic species. This ICM divides the cell into two parts: the paryphoplasm and the pirellulosome. The pirellulosome contains all the ribosomes, whereas the paryphoplasm is ribosome free. This type of cell compartmentalization occurs in members of Verrucomicrobia. More than seven Planctomycetaceae type species have been reported from different genera, namely, *Blastopirellula* [6], [7], *Gemmata* [8], *Isosphaera* [9], *Pirellula* [10], [11], *Rhodopirellula* [6], *Schlesneria* [13] and *Singulisphaera* [15]. Only *Gemmata obscuriglobus* exhibits a double-membrane ICM, whereas other type species have a single membrane. Phylogenetic studies on the conserved regions in the ribosomal RNA sequences and whole proteomes suggest that Planctomycetaceae are closely related with Verrucomicrobia and Chlamydiae, which are all members of the Planctomycetaceae*–*Verrucomicrobia*–*Chlamydiae (PVC) superfamily [18]. Jun et al. [19] proposed that Planctomycetaceae should be classified between bacteria and archaea based on their evolutionary relationship. Brochier and Philippe [20] found that Planctomycetales emerged at the base of the bacterial branch, according to the conserved positions in ribosomal RNA.

To date, overviews on the genomic characteristics of planctomycete are insufficient. As the fast development of sequencing technology, the foundations are starting to be laid for comparative genomics to assist in the interpretation of planctomycete cell biology. In this study, we attempted to unveil the relationships among Planctomycetaceae species and to explore their evolutionary status among organisms using whole-genome analysis.

## Results and Discussion

### Overview of Planctomycetaceae Genomes

We previously sequenced three Planctomycetaceae type strains (*Singulisphaera acidiphila* DSM 18658^T^, *Schlesneria paludicola* DSM 18645^T^, and *Zavarzinella formosa* DSM 19928^T^) [14]. These strains were isolated from acidic wetlands in northern Russia (http://www.dsmz.de/). The whole genomes of type species from six other genera are available in NCBI (Table 1). More than two type strains of the genus *Planctomyces* were also sequenced. We performed a preliminarily exploration of the resistance genes in 11 Planctomycetaceae genomes (Supplementary Fig. S1) [21].

**Table 1:** Genome survey of 11 strains in family Planctomycetaceae.

Species	Accession number(GenBank)	Genome size(Mb)	GC content(%)	Genecount	Proteincount
*Planctomyces limnophilus* DSM 3776^T^	NC_014148	5.46	53.70	4,373	4,258
*Isosphaera pallida* DSM 9630^T^	NC_014962	5.53	62.40	3,823	3,722
*Planctomyces brasiliensis* DSM 5305^T^	NC_015174	6.01	56.40	4,865	4,750
*Pirellula staleyi* DSM 6068^T^	NC_013720	6.20	57.50	4,825	4,717
*Blastopirellula marina* DSM 3645^T^	AANZ00000000	6.65	57.00	6,079	6,025
*Rhodopirellula baltica* DSM 10527^T^	NC_005027	7.15	55.40	7,404	7,325
*Planctomyces maris* DSM 8797^T^	NZ_ABCE00000000	7.78	50.50	6,530	6,480
*Schlesneria paludicola* DSM 18465^T^	AHZR00000000	8.70	56.00	8,626	8,626
*Gemmata obscuriglobus* DSM 5831^T^	ABGO00000000	9.16	67.20	8,080	7,989
*Singulisphaera acidiphila* DSM 18658^T^	AHZQ00000000	9.73	63.01	8,972	8,972
*Zavarzinella formosa* DSM 19928^T^	AIAB00000000	10.09	59.60	10,112	10,112

**Figure 1:**
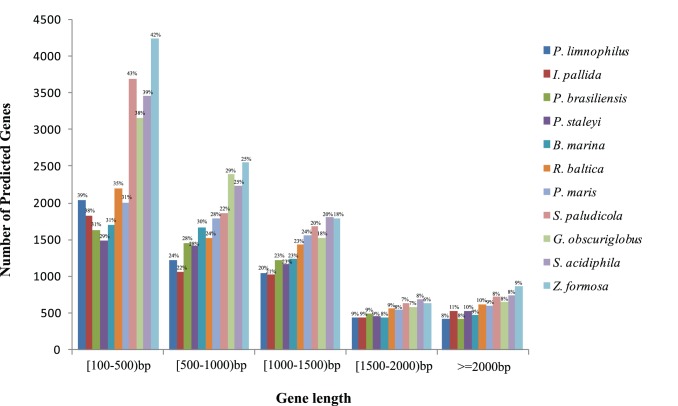
Gene length distribution in the 11 Planctomycetaceae genomes. The values above each bar (in percent) were calculated as the number of genes that are of specified length divided by the number of total genes in each Planctomycetaceae genome.

**Figure 2:**
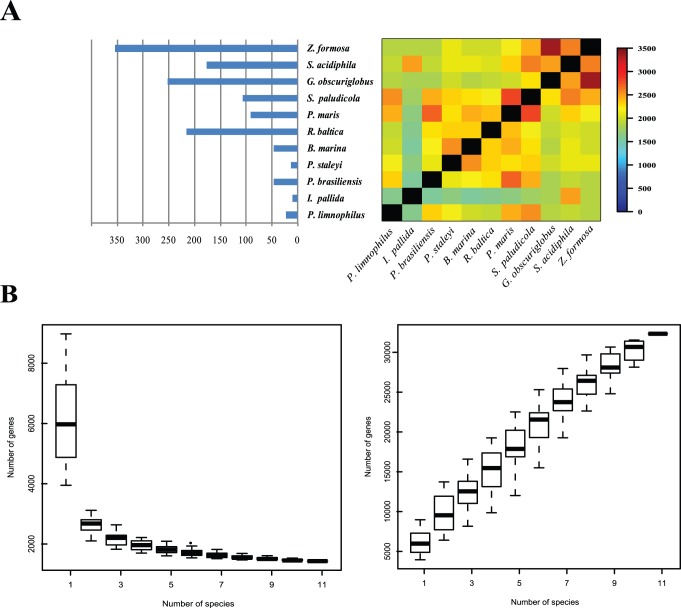
Gene family clusters and core genome/pan genome analyses based on whole-genome sequences. (A) The histogram reveals the number of unique gene families in each of the 11 Planctomycetaceae strains. The color bar on the right shows the number of common gene families between crossed strains. (B) The box plot at the left indicates the number of core genes that correspond with the strain genomes. The box plot at the right indicates the pan gene numbers that correspond with the strain genomes. The box in the figures indicates half the distribution of values in the random sampling; the bold line in box indicates the median.

The Planctomycetaceae genomes are over 5 Mb long and possess more than 3,700 coding genes (CDSs), which are relatively larger than those of most bacteria. The genome size of *Z. formosa* is 10.09 Mb with 10,112 CDSs, similar to the ∼12 Mb genome of *Saccharomyces cerevisiae* [22], but with twice the number of CDSs. Except for a small subset of individuals, the number of CDSs is roughly proportional to the genome size (Table 1). Deviations were inevitable because 8 of the 11 genomes were obtained from NCBI and they may contain gaps and sequencing errors. Furthermore, the C-value paradox should be considered. From fig. 1, in the 11 genomes, the length of genes are mostly less than 1,000 bp. Remarkably, in the range of [100–500) bp, the drastic increase of genes in the larger genomes was observed. *G. obscuriglobus* had the highest GC content, ranging from 50.50% to 67.20% in the 11 Planctomycetaceae genomes (Table 1). Planctomycetaceae has more than 30,000 pan-genes and 832 core gene clusters with 9,928 genes. Jogler et al. [23] used the 8 genomes excluding the 3 genomes sequenced by us, and found that the planctomycetal core genome led to 114 predicted protein clusters containing 2,908 proteins from all eight analyzed planctomycetes after the in silico subtraction of *E. coli* and *B. subtilis* genomes with all-against-all BLASTP approach (with stringent filters: coverage higher than 60% and below the E value cutoff of 1e −5 were taken into account). Proteome-based analysis of the predicted protein families in the 11 genomes (Fig. 2A and Supplementary Table S1) showed that *Z. formosa* and *G. obscuriglobus* share 3,344 common protein families. Furthermore, *P. maris* and *S. paludicola* share 2,821 protein families, *P. maris* and *P. brasiliensis* share 2,684 protein families. *Z. formosa* possesses the highest number of unique families with 1,092 proteins in 355 protein families, which is followed by *G. obscuriglobus* with 745 proteins in 252 families and *Rhodopirellula baltica* with 356 proteins in 216 families. In addition, numerous orphan proteins excluded from the final clusters and paralogs found in the predicted proteomes are responsible for the large size of Planctomycetaceae genomes. To confirm their relationships, we analyzed the tetranucleotide frequencies and the average nucleotide identity (ANI) [24], [25] of the 11 genomes (Supplementary Figs. S2A and S2B).

### Evolutionary Research on Planctomycetaceae

Fuerst et al. [26] reconstructed evolutionary models of the NE to illustrate the resemblance between Planctomycetales and fungi in terms of their compartmentalization. Jun et al. [27], [28] used an alignment-free method based on whole proteome FFPs (or monomers) to compare the FFPs of 900 proteomes from 26 phyla. The results revealed that Planctomycetaceae was found between bacteria and archaea in the phylogenetic trees. Moreover, the universal primers of bacterial 16S rRNA genes may not be effective for polymerase chain reaction amplification of the corresponding planctomycete genes [29]. That is, certain species in Planctomycetales may have been overlooked by the metagenomic analyses. Furthermore, the 5S rRNAs of Planctomycetales were shorter (109 nt to 111 nt) than the “minimal” length found in other prokaryotes (118 nt) and uniquely lacked an insertion at position 66. Brochier and Philippe [20] reanalyzed the bacterial phylogeny based on rRNA sequences using the “slow–fast” method [30], which is more reliable for identifying the most slowly evolving positions that are less affected by artifacts. Their study found that the Planctomycetales family might have emerged at the base of the bacteria branch.

**Figure 3:**
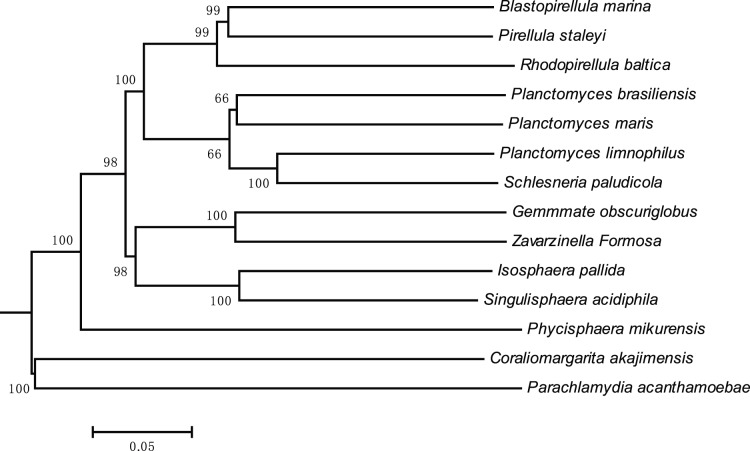
Phylogenetic tree constructed using proteomes predicted from 11 genomes of Planctomycetaceae. The rooted tree generated in MEGA 4.0 with the concatenated alignments of the conserved regions of the orthologous core protein sequences, topology of which illustrates the relationships among Planctomycetaceae species and related taxa. Bootstrap values (in percent) of 1000 simulations are indicated at all branches. Bar represents 0.05 amino acid substitutions per site. *Coraliomargarita akajimensis*, *Parachlamydia acanthamoebae*, and *Phycisphaera mikurensis* were selected as outgroups [31].

A core-gene tree was created based on the concatenated alignments of the core genes and only the conserved regions of the orthologous core protein sequences were used to construct this genome-wide tree. We used protein sequences rather than nucleic acid sequences to minimize the possible influence of base composition and codon preference biases on phylogenic construction. The reasonable branching of the phylogenetic trees was consistent with the different physiologic and biochemical features of Planctomycetaceae species (Fig. 3). For instance, both *R. baltica* and *Blastopirellula marina* live in marine environments with high NaCl concentrations. In addition, *Pirellula staleyi*, *R. baltica*, and *B. marina* are strain of genera from the “*Pirellula* group,” which are closely similar in morphology and physiology despite having less than 90% similarity of their 16S rRNA gene sequences [6]. Sequence alignment of the 16S rRNA genes confirmed that Planctomycetaceae is present in environmental correlation groups [31]. Therefore, we selected three closely related species that belonged to environmental correlation groups as outgroups to determine the evolutionary status of Planctomycetaceae. Results indicate that the predicted proteomes accurately represent the phylogenetic relationships among members of Planctomycetaceae.

### Gene Function Annotation and Metabolic Pathways Reconstruction

**Figure 4:**
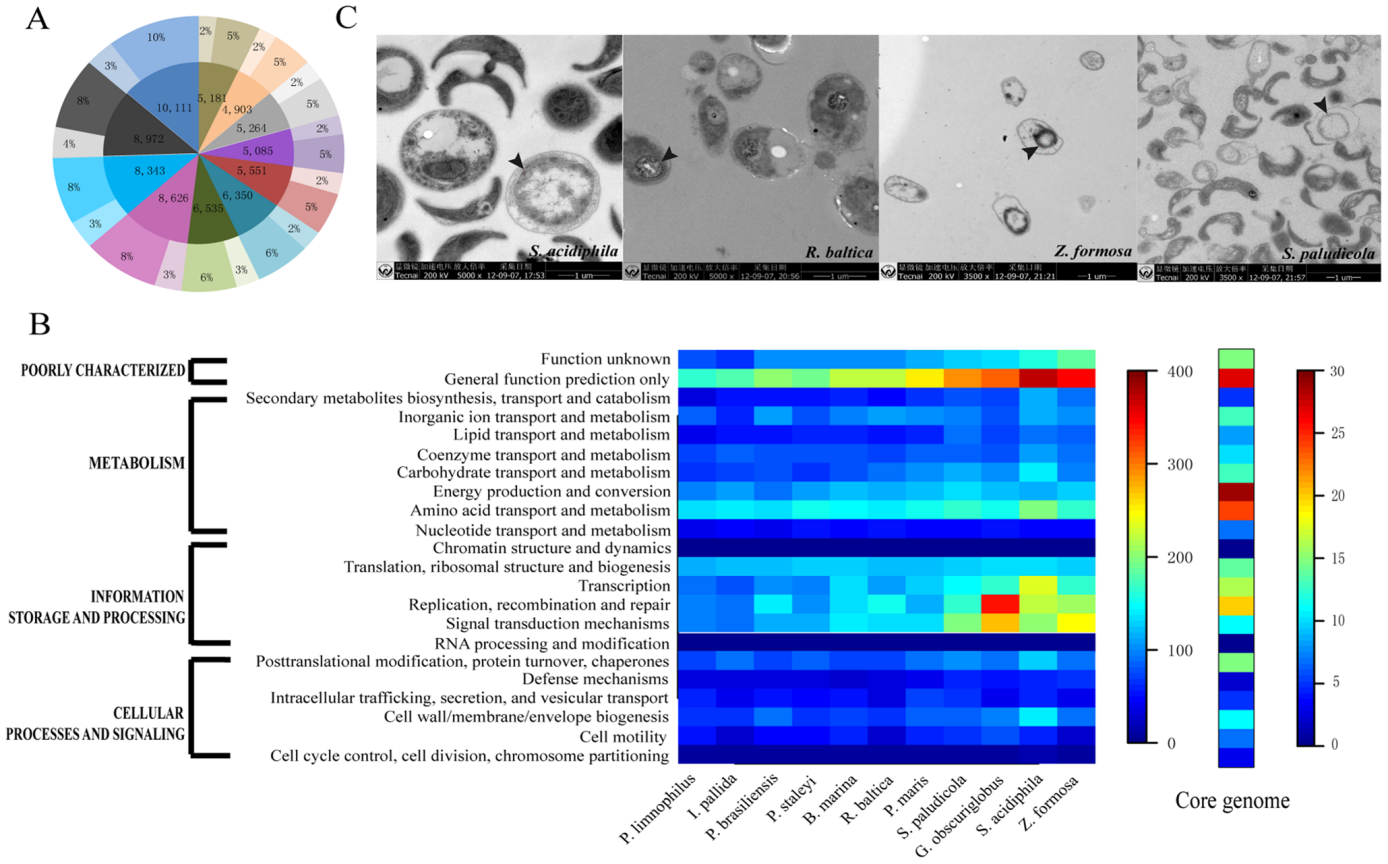
Gene annotation. (A) The inner ring: number of genes in the genomes; The outer ring: characterized/uncharacterized genes. (B) Gene function classification based on COG annotation. Heatmap at the left shows the functional prediction of 11 Planctomycetaceae genome. The core genome consists of the nonredundant core protein families. Heatmap at the right shows the functional prediction of the core genome. The color bar shows gene numbers. (C) ICM structure of four Planctomycetaceae species. These pictures shows the the ICM structure of four Planctomycetaceae species, namely *S. acidiphila*, *R. baltica*, *Z. formosa*, and *S. paludicola* (left to right), which were observed under transmission electron microscopy.

We annotated the gene function of the 11 Planctomycetaceae genomes with COG databases and predicted their metabolic pathways using the KEGG database. To our surprise, only 29.28% of the total genes in the 11 genomes have been characterized, numerous genes remain unclear (Fig. 4A). Jogler et al. [23] reported that more than 55% of the predicted proteins are of unknown function in each genome on average in Planctomycetaceae. In the 11 sequenced species, only *R. baltica* (originally classified as *Pirellula* sp. str. 1) [32] has been described in detail in the literature. *P. staleyi* and *P. limnophilus* genomes were described in summary form only [10], [12]. The function could be predicted for only 32% of the ORFs in *R. baltica*; although around 54% of ORFs with a predicted function were in *P. staleyi* and *P. limnophilus*, hypothetical proteins are a feature of planctomycetes, and this remains a challenge for understanding the molecular cell biology of these bacteria [18]. The same case occurred in their core genome. We obtained 832 core families, only 255 of which are characterized in the COG database. For the pan genome, genes related with general function is dominant except *G. obscuriglobus*, genetic information of which is the most prominent. But the situation is different in their core genome, in which the energy production and conversion is dominant, and then general function. Overall, Genes involving general function, amino acids transport and metabolism, replication/recombination and repair, signal transduction mechanisms and translation/ribosomal structure/biogenesis governed in either pan genome or core genome of Planctomycetaceae (Fig. 4B). Given that most scientists pay more attention to the unique cell biological characteristics, few focus on the whole metabolic pathways of Planctomycetaceae family thus far. Such as, identification protein domains likely to be involved in morphogenesis, cell Division and signal transduction in Planctomycetes by comparative genomics [23]; the endocytosis process in *G. obscuriglobus* with eukaryotic membrane coat (MC)-like-protein-encoding genes [33]; the unusual peptidoglycan (PG)-free cell wall and ICM structure [18] and so on. Actually, the whole metabolic pathways analysis can help us explain the whole biological features well and provide more information to our research. Since *R. baltica* is the first planctomycete to be genome completely sequenced and one of the best-studied planctomycetes on the proteomic level, we mainly focused on it for the illustration of the subsequent metabolic pathways research. Glockner et al. [32] partly predicted the metabolic pathways of *R. baltica* in bioinformatical ways, for instance, C1 metabilism and Entner-Doudoroff pathway. Hieu et al. [34] experimentally identified 1,267 nonredundant proteins (accounting for 17.3% of the total putative protein-coding ORFs) from *R. baltica* proteome ananlysis, and reconstructed a rough depiction of house-keeping metabolic routes of carbohydrate, amino acid, nucleic acid and fatty acid metabolism pathways of *R. baltica* from 668 functionally assigned proteins.

In this study, the 11 Planctomycetaceae genomes contained 231 predicted metabolic pathways and 15,061 enzymatic reactions, among which 1,280 are in *P. limnophilus*, 1,264 are in *I. pallid*, 1,353 are in *P. brasiliensis*, 1,340 are in *P. staleyi*, 1,305 are in *B. marina*, 1,391 are in *R. baltica*, 1,371 are in *P. maris*, 1,445 are in *S. paludicola*, 1,374 are in *G. obscuriglobus*, 1,565 are in *S. acidiphila*, and 1,373 are in *Z. formosa* (Supplementary Table S2). Although the function of some genes in the metabolic pathways may be replaced and cannot be identified during evolution, core metabolic pathways can provide relatively accurate genetic information inherited from their ancestor. In all, 123 metabolic pathways in KEGG pathway database responded to all the 11 genomes of Planctomycetaceae. Their ancestors should have a comprehensive metabolism based on these pathways (Supplementary Fig. S4). Glycolysis/gluconeogenesis, the citrate cycle (TCA cycle), the pentose phosphate pathway, and oxidative phosphorylation are the metabolic junctions of energy generation and intermediate compounds, which was supported in the former proteomic research of *R. baltica* [34], [35]. Gade et al. [35] found that *R. baltica* cells could be adapted to growth with eight different carbohydrates: glucose, ribose, N-acetylglucosamine, xylose, maltose, lactose, melibiose, and raffinose, respectively. We found these species also have the potential of using fructose, galactose, starch, sucrose, and mannose. Moreover, they can generate energy through sulfur and nitrogen metabolism. Some photosynthesis- and carbon fixation–related genes were also found. Furthermore, almost all 20 amino acid metabolic pathways were detected, which agreed with the *R. baltica*’s proteomic study [34]. Remarkably, nearly all the genes for the synthesis of the seven amino acids essential to humans were found. We infer that these species are likely able to synthesize these amino acids, namely, valine, leucine, phenylalanine, tyrosine, lysine, tryptophan, and isoleucine. Kerger et al. [36] reported the presence of hydroxyl fatty acids in planctomycetes and deduced that they originate from lipopolysaccharides (LPS). Schlesner et al. [6] concluded that the absence of measurable amounts of hydroxyl fatty acids indicates the absence of significant amounts of lipopolysaccharides in the cell wall even though the organisms are Gram negative. In our study, some of the peptidoglycan biosynthesis genes and most of the *Escherichia coli* lipopolysaccharide biosynthesis genes were detected in all 11 genomes (Supplementary Fig. S3).

Most unexpectedly, the 11 species can synthesize unsaturated fatty acids and complex lipids such as steroids [37]. In particular, *Gemmata* spp. can synthesize C_30_ sterols such as lanosterol, which are speculated to regulate membrane fluidity in the characteristic internal membranes of planctomycetes [18]. Although *R. baltica* is not capable of utilizing methanol, methylamine, or methylsulfonate [20], we detected biosynthetic genes for zeatin, steroids, steroid hormones, carotenoids, ubiquinones, and other terpenoid quinones in all 11 genomes. In addition, we found an intact mevalonate pathway conserved among the 11 species that produces the precursor of the terpenoid backbone, which indicates that Planctomycetaceae species are capable of synthesizing complex compounds. Schlesner et al. [6] found that the absorption spectrum of a methanol extract peaks at 495 nm and forms two shoulders at 460 and 520 nm, which are similar to that of carotene. However, few homologous genes related to steroid and carotene biosynthesis were found, which illustrates that the conserved domains of functional genes have been replaced during evolution or that these species use new biosynthetic pathways.

Leary et al. [38] cloned the *rpoN* gene from *P. limnophilus* that encodes alternative sigma factor σ^54^, which is involved in diverse metabolic functions such as nitrogen fixation, hydrogen metabolism, and degradation of aromatic compounds, and they demonstrated complementation in a *Salmonella typhimurium* mutant. Analysis of the core metabolic pathways of the 11 species revealed that all of them shared metabolic pathways for degrading diverse toxic compounds, including ethylbenzene, aminobenzoate, naphthalene, chloroalkane, polycyclic aromatic hydrocarbon, toluene, bisphenol, benzoate, chlorocyclohexane, and chlorobenzene (Supplementary Fig. S3). This finding indicates that these species may have potential applications in environmental management. We marked the metabolic pathways for degrading aromatic compounds with bold rectangle in supplementary fig. S3. On the other hand, these species possess genes for synthesizing streptomycin and other antibiotics, such as tetracycline, ansamycins, vancomycin, novobiocin, butirosin, and neomycin. Furthermore, we also found biosynthetic genes for alkaloids, which are mostly toxic to humans.

Despite the ABC transporter system has been found in *R. baltica* [34], [35], F-type ATPases and ATP-binding cassette transporters were detected in all the 11 species (Supplementary Fig. S3). After analyzing the core metabolic pathways, we found a high level of two-component systems, which verify that these species possess complex regulatory systems for adapting to their physiological and functional complexity. Actually, Planctomycetaceae species have an ICM structure [18], which is unusual in prokaryotic and eukaryotic species (Fig. 4C). However, its composition and function remain unclear. We inferred that two-component systems widely regulate the life cycle by phosphorylation/dephosphorylation based on a phosphotransferase system (PTS) in the 11 species.

### Structural Variations (SVs) and Genomic Islands (GIs) in the Five Complete Planctomycetaceae Genomes

**Figure 5:**
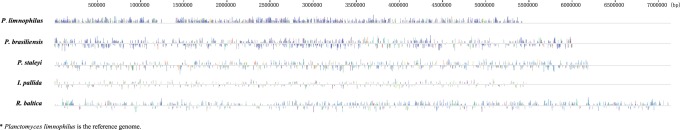
Collinearity of the five completed Planctomycetaceae genomes. *P. limnophilus* was set as the reference genome. Different colored lines in each genome indicate its similarity profile. The height of the similarity profile corresponds to the average level of conservation in that region of the genome sequence and was calculated to be inversely proportional to the average alignment column entropy over a region of the alignment. Parts of the similarity plot which are colored mauve are conserved among all the five genomes. The other colors shows the similarity of segments not in all the five genomes, but they are too small to notice in a whole-genome view. The similarity profile above the centerline of the aligned region is in the forward orientation relative to the first genome sequence, and profiles below the centerline indicate regions that align in the inverse orientation. Areas that are completely white were not aligned and probably contain sequence elements specific to a particular genome. The Mauve software was used for this analysis [55]. The seed (minimum match size) we used was 15 mer.

**Figure 6:**
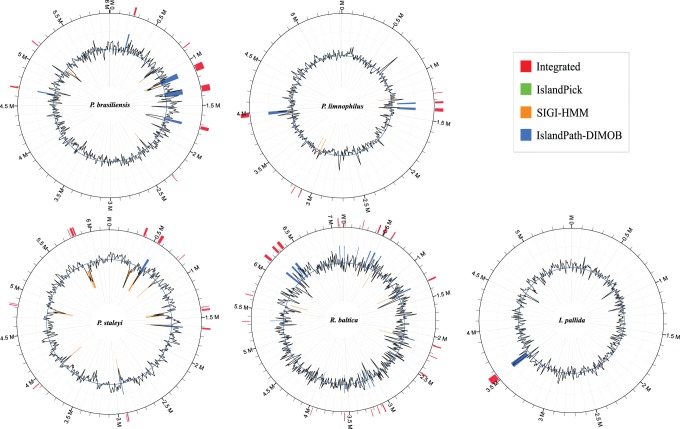
Overview of genomic islands in the five completed genomes. GI prediction was performed by integrating the following software: SIGI-HMM, IslandPath-DIMOB and IslandPick. The prediction was implemented in Web server IslandViewer (http://pathogenomics.sfu.ca/islandviewer/query.php) [44].

Many factors are responsible for the genome expansion or shrink, including homologous recombination, horizontal gene transfer (HGT), convergent/divergent evolution and specialization [39], [40]. Glockner et al. [32] found that multicopy genes make up for about 25.4% of the *R. baltica* genome sequence, which is less than the 30% reported for *Bacillus subtilis* [41] and the 29% calculated for *Escherichia coli* K-12. Therefore extensive gene duplication is not the reason for the large genome size of *R. baltica*. Gade et al. [35] cultivated *R. baltica* cells with 8 different carbohydrates respectively and profiled with 2-DE for changes in protein patterns. They found that most genes not organized in clusters are functionally related and coordinately regulated, which is in agreement with the previous in silico observation that functionally related genes are often not clustered in operon-like structures in the 7.145 Mb genome of *R. baltica* [32]. This contrasts the genetic organization known from standard bacteria such as *E. coli* or *B. subtilis*, both having considerably smaller genome sizes than *R. baltica*. Apparently, the number of genes organized in clusters decreases with increasing genome size [42]. In addition, a large genome with an expanded genetic capability might be a prerequisite for environmental adaptability, as already discussed for the genome of *Pseudomonas aeruginosa* [43]. In this case, it is necessary to investigate the genome arrangement and genomic islands in the 11 genomes. We found that numerous unique segments were inserted between homologous blocks, which indicate that homologous recombination and HGT facilitate the divergent evolution of these genomes (Fig. 5). On the other hand, we selected the 5 completed genomes of *P. limnophilus*, *P. brasiliensis*, *P. staleyi*, *I. pallida*, and *R. baltica* to predict accurately the GIs and SVs. GI prediction was performed by integrating the softwares SIGI-HMM, IslandPath-DIMOB and IslandPick (Fig. 6) [44]. In a recent study, the SIGI-HMM method was shown to have the highest precision and overall accuracy among six tested sequence composition GI prediction methods [45]. Based on the prediction results from SIGI-HMM, the proportion of GIs was less than 5.0% of the five genomes and most of the GIs originated from Actinobacteria, and then, from *Bacillus*, *Nitrospira*, *Bacteroides*, Chloroflexi, Gammaproteobacteria in the five completed genomes. Up to 355 genes were in the *R. baltica* GIs, 65 were in the *P. limnophilus* GIs, 161 were in the *P. staleyi* GIs, 234 were in the *P. brasiliensis* GIs, and 58 were in the *I. pallid* GIs. However, most of them remain uncharacterized (Supplementary Table S3). We found LPS biosynthesis–related proteins in the GIs of *P. limnophilus* and *R. baltica*, streptomycin biosynthetic proteins and its operon regulatory protein in the GIs of *R. baltica*, CRISPR-associated proteins in *I. pallid*, cytochrome P450 genes in *P. brasiliensis* and *P. staleyi*, bifunctional DNA primase/polymerase in *P. brasiliensis* and *I. pallid*, sigma-70 family RNA polymerase sigma factor in *P. limnophilus*, and WD-40 repeat-containing protein in *P. staleyi*.

**Figure 7:**
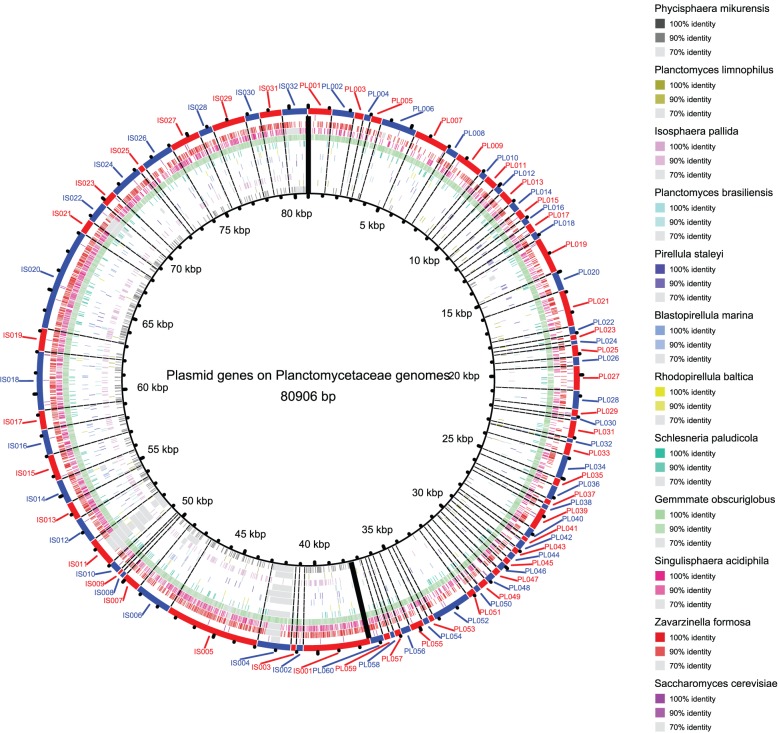
Plasmid integration in the 11 genomes. PL and IS represents the plasmid reported in *P. limnophilus* and *I. pallid* respectively. The outermost ring shows the two plasmids’ genes which divided by bold lines. The colors in each genome ring accord with the identity illustrated in the legend. The 11 Planctomycetaceae genomes were arranged according to their genome sizes, the outer the larger. *Phycisphaera mikurensis* and *Saccharomyces cerevisiae* were used as a control.

To further explore the HGT, we examined the plasmid integration in all 11 Planctomycetaceae genomes (Fig. 7). Only two plasmids were reported in Planctomycetaceae, from *P. limnophilus* and *I. pallid*, and they are available in NCBI. In addition, we selected the closely related *Phycisphaera mikurensis* and the low-grade fungus *Saccharomyces cerevisiae* as controls. Strikingly, the *I. pallid* plasmid genes exhibited high identity with those in the genomes of *P. mikurensis*, *S. paludicola*, *G. obscuriglobus*, *S. acidiphila*, and *Z. formosa*, whereas *P. limnophilus* plasmid genes exhibited high identity with those in the genomes of *S. paludicola*, *G. obscuriglobus*, *S. acidiphila*, and *Z. formosa*. However, the plasmid genes of *I. pallid* and *P. limnophilus* were not detected in the *S. cerevisiae* genome. Larger genome sizes seemed to be correlated with higher coverage and with homology of plasmid genes. The results indicate that the plasmid integration in the 11 genomes may also lead to larger genome sizes; moreover, it reflects that the potential shuttle ability of Planctomycetaceae plasmids among their close relatives.

### Conclusions

Worldwide interest in the unique cellular biological features and structure in Planctomycetaceae has increased considerably, especially in evolutionary research. Exploring the potential genetic information in this special groups based on comparative genomic ways is essential for conducting the later researches. In this context, we mainly researched the genomic features and metabolic pathways with 11 type strains from different genus of family Planctomycetaceae. The genome-scale information we obtained combined with the already developed physiologic, genetic, and metabolic approaches will increase our knowledge about family Planctomycetaceae and may help us establish strategies for developing them for human applications. In this way, we could learn much about their relationships and potential biological systems of these species. Acquiring of these results were made possible by the use as reference to subsequent study. Further, we might have broader horizon to discover new features in these species responding to their attractive ICM structure, which is significant in evolution from prokaryotes to eukaryotes.

## Materials and Methods

### Source of Genomes

The genomes of eight species from family Planctomycetaceae were downloaded from GenBank (Table 1), namely, *P. limnophilus*, *I. pallida*, *P. brasiliensis*, *P. staleyi*, *B. marina*, *R. baltica*, *P. maris*, *G. obscuriglobus.* The genomes of *Schlesneria paludicola*, *Singulisphaera acidiphila*, and *Zavarzinella formosa* were sequenced in our previously study [14].

### Ortholog Retrieval

Orthologs were determined using OrthoMCL [46]. This program first performs an all-against-all BLASTp in BLAST 2.2.25. OrthoMCL then converts the reciprocal BLAST p-values to a normalized similarity matrix that is analyzed using the Markov Cluster algorithm (MCL). The MCL analysis yielded numerous clusters, with each cluster containing a set of orthologs and/or recent paralogs. The BLAST e-value cutoff was ≤10^−5^; the other parameters were set to their default values.

### Core/Pan Genome Analysis

The pan genome of Planctomycetaceae was constructed by selecting one genome as the origin and then gradually adding the other genomes individually. The final pan-genome was obtained by discarding redundant clusters. A set of identity matrices generated through BLAST analyses of all genomes and then selecting the nonredundant clusters conserved in all matrices as the core genome. The parameters used in core genome/pan genome analyses were as follows: e-value ≤10^−5^, identity ≥50%, and coverage ≥50%.

### Calculation of ANI (ANIb) and Tetranucleotide Frequencies

ANI values were calculated as described by Goris et al. [47] (Fig. S2A). The tetranucleotide frequencies and correlation coefficients were calculated based on the algorithm described by Teeling et al. [48] (Fig. S2B).

### Construction of Phylogenetic Trees Based on the Predicted Whole Proteomes from the Genomes

Several steps were involved in the phylogenetic tree construction based on the 11 predicted proteomes of Planctomycetaceae. First, performing all-against-all BLASTp using the 11 Planctomycetaceae predicted proteomes with BLAST 2.2.21 and setting appropriate identity and coverage cutoffs to extract their core genome; Then, implementing multiple sequence alignment in MUSCLE 3.8.31 [49] with the core genome; Subsequently, generating tree files in TREEBEST 1.9.2 using the neighbor-joining method; Finally, constructing phylogenetic trees in MEGA 4.0 [50] with the bootstrap value of 1000. A core-genome tree was created based on the concatenated alignments of the core proteins and only the conserved regions of the orthologous core protein sequences were used to construct this genome-wide rooted tree, up to 97 core proteins were obtained using a BLAST query, with e-value ≤10^−5^, identity ≥30%, and coverage ≥60%. *Coraliomargarita akajimensis*, *Parachlamydia acanthamoebae*, and *Phycisphaera mikurensis* were selected as outgroups according to the 16S rRNA classification [23], [31].

### Annotation

Functions of genes were assigned according to the best match of the alignments using BLASTp (E-value ≤10^−5^) searching against the SwissProt and Uniprot databases (Release 15.10) [51]. The motifs and domains of proteins were determined using InterProScan (Version 4.5) [52] against protein databases. Furthermore, all proteins were aligned against KEGG (Release 48.2) [53] proteins. If the best hit of the proteins in any of these processes was “function unknown,” “putative,” the second best hits were used to assign function until no more hits met the alignment criteria.

### Metabolic Reconstruction

We reconstructed the metabolic pathways of the 11 species using the web server Ipath (http://pathways.embl.de/) [54] with their assigned K numbers in KEGG Orthology system.

### Collinear Analysis

Collinearity analysis of the five completed genomes was implemented in Mauve [55].

### Genomic Island Search

Genomic islands were searched using the web server IslandViewer (http://pathogenomics.sfu.ca/islandviewer/query.php) [44], which integrates the three mainstream methods: SIGI-HMM, which focuses on codon usage; IslandPath-DIMOB, which focuses on dinucleotide and mobile elements; and IslandPick, which focuses on comparative genomic GI prediction. A plasmid integration survey of the 11 genomes was carried out with software BRIG [56], which run local BLAST-2.2.27+ and generated the figure in java.

## Supporting Information

Figure S1 **Resistance genes in the 11 Planctomycetaceae genomes.**
(TIF)

Figure S2 **Calculation of ANI (ANIb) and tetranucleotide frequencies.**
(TIF)

Figure S3 **Metabolic analysis of the 11 Planctomycetaceae species.** Color of the heatmap box means gene number, which indicate in the color bar at the right of the heatmap; the colored box in legend at the left shows the same meaning with the colored text in the heatmap.
(PDF)

Figure S4 **Reconstruction of the metabolic pathways of the 11 Planctomycetaceae genomes.** Reconstruction of the metabolic pathways was implemented in the KEGG orthology system. The common metabolic pathways of the 11 Planctomycetaceae genomes are in green color, and their dispensable metabolic pathways are in red.
(TIF)

Table S1 **Protein families among the 11 Planctomycetaceae genomes.**
(XLSX)

Table S2 **Gene distibution in the metabolic pathways of the 11 Planctomycetaceae genomes.**
(XLSX)

Table S3 **Genes in the GIs of the five completed Planctomycetaceae genomes.**
(XLSX)
