# Supplementary figures and images for: Genomic Evolution of 11 Type Strains within Family Planctomycetaceae
Source: PLoS One. 2014 Jan 29;9(1):e86752. doi: 10.1371/journal.pone.0086752 (PMC3906078; doi:10.1371/journal.pone.0086752)

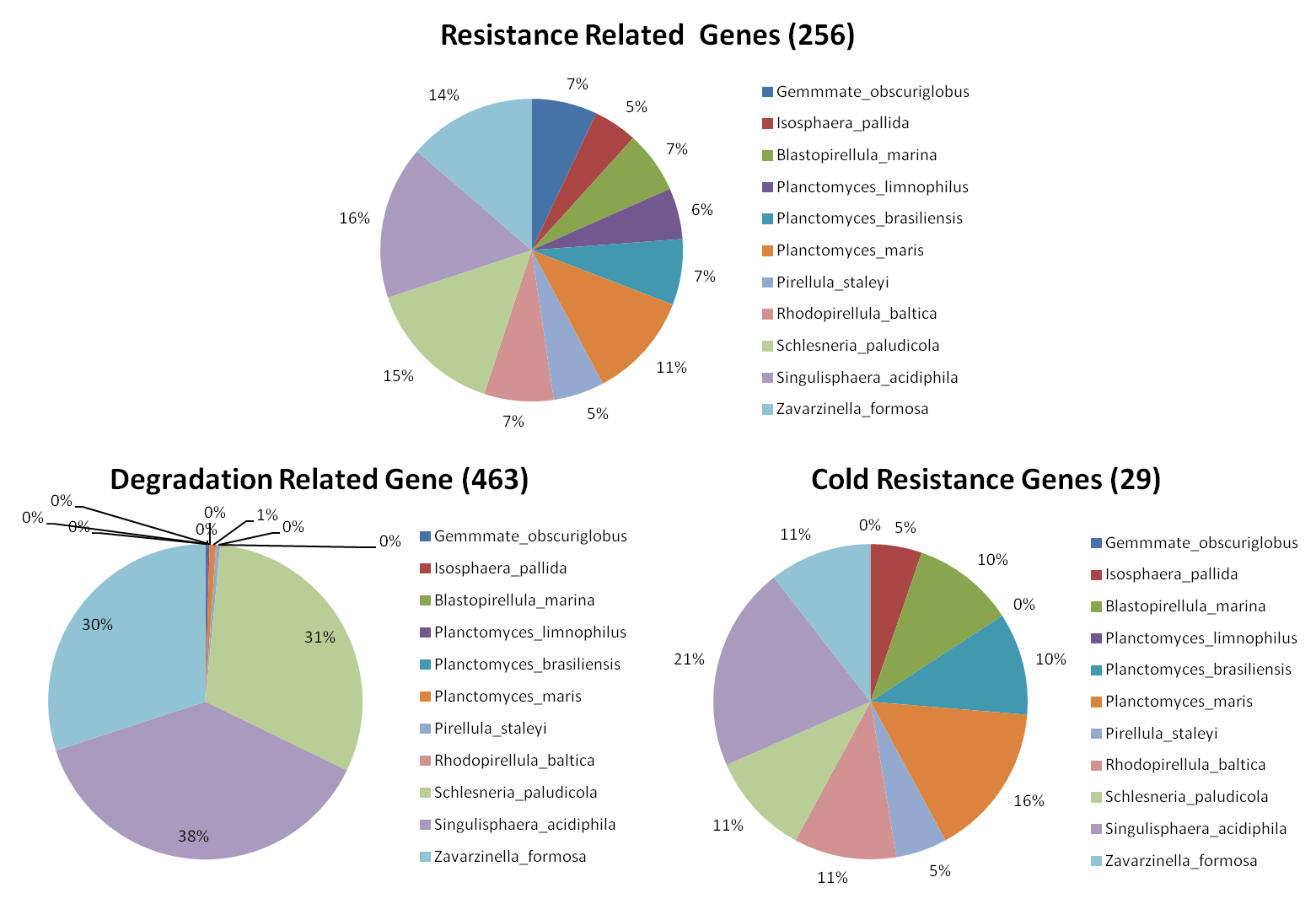

Supplement: Figure S1 — Resistance genes in the 11 Planctomycetaceae genomes. (TIF) [file pone.0086752.s001.tif]

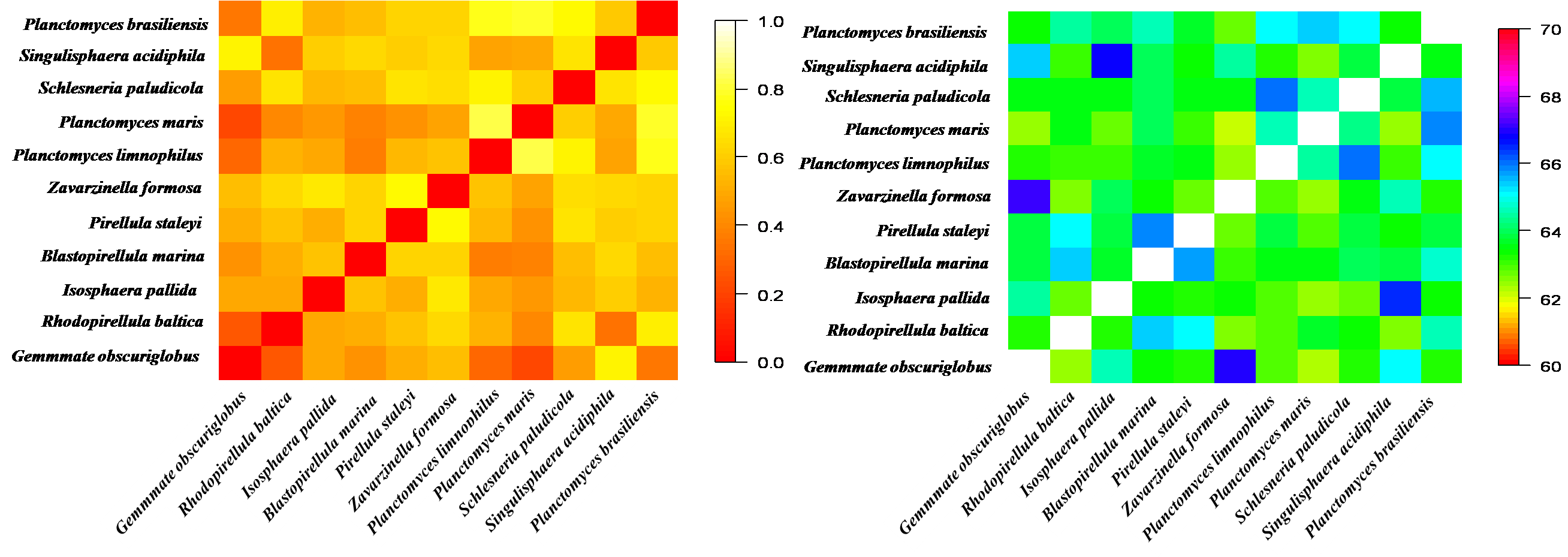

Supplement: Figure S2 — Calculation of ANI (ANIb) and tetranucleotide frequencies. (TIF) [file pone.0086752.s002.tif]

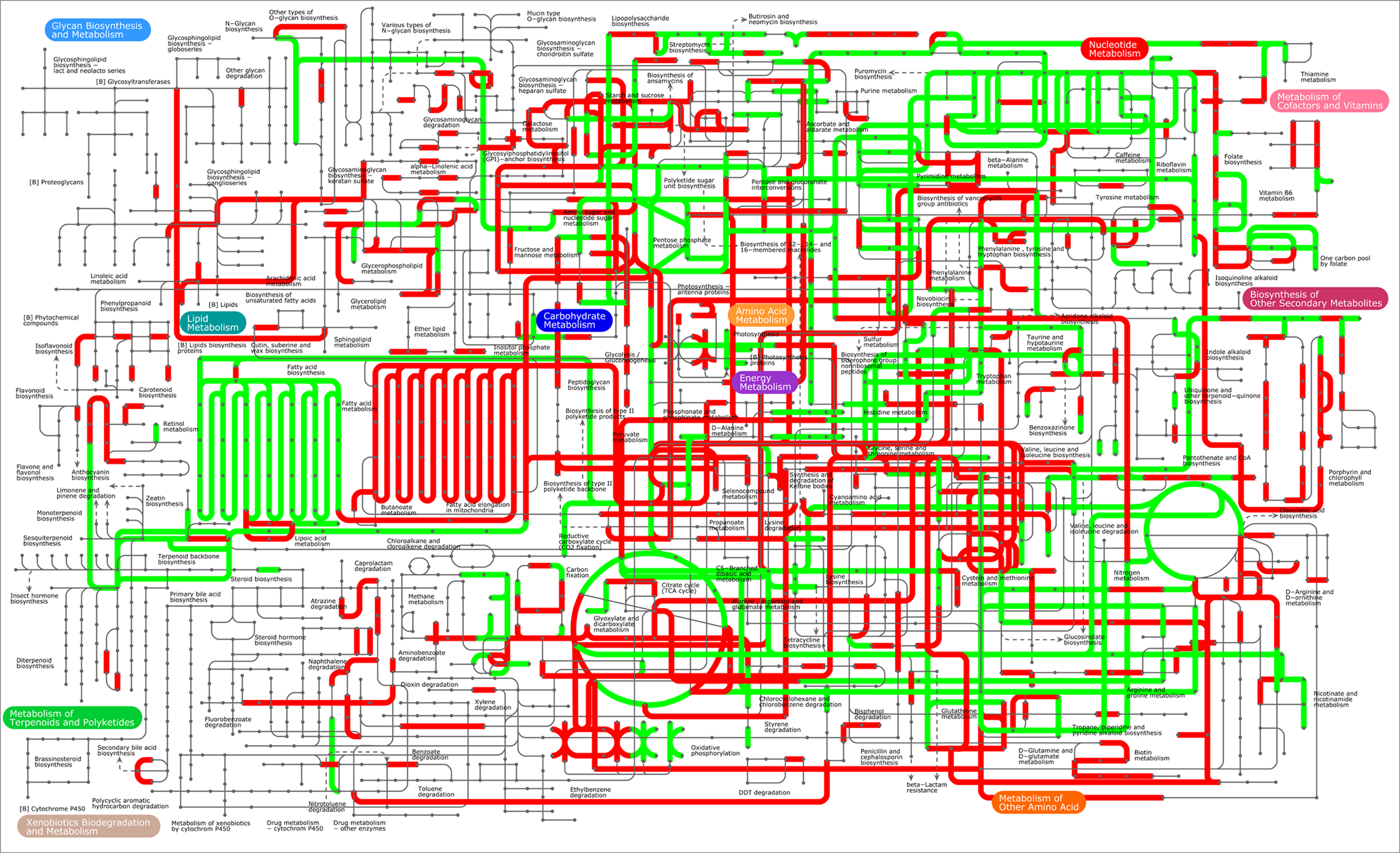

Supplement: Figure S4 — Reconstruction of the metabolic pathways of the 11 Planctomycetaceae genomes. Reconstruction of the metabolic pathways was implemented in the KEGG orthology system. The common metabolic pathways of the 11 Planctomycetaceae genomes are in green color, and their dispensable metabolic pathways are in red. (TIF) [file pone.0086752.s004.tif]
