# Supplementary material for: Genomic Evolution of 11 Type Strains within Family Planctomycetaceae
Source: PLoS One. 2014 Jan 29;9(1):e86752. doi: 10.1371/journal.pone.0086752 (PMC3906078; doi:10.1371/journal.pone.0086752)

KEGG matabolic pathways of 11 *Planctomycetaceae* species

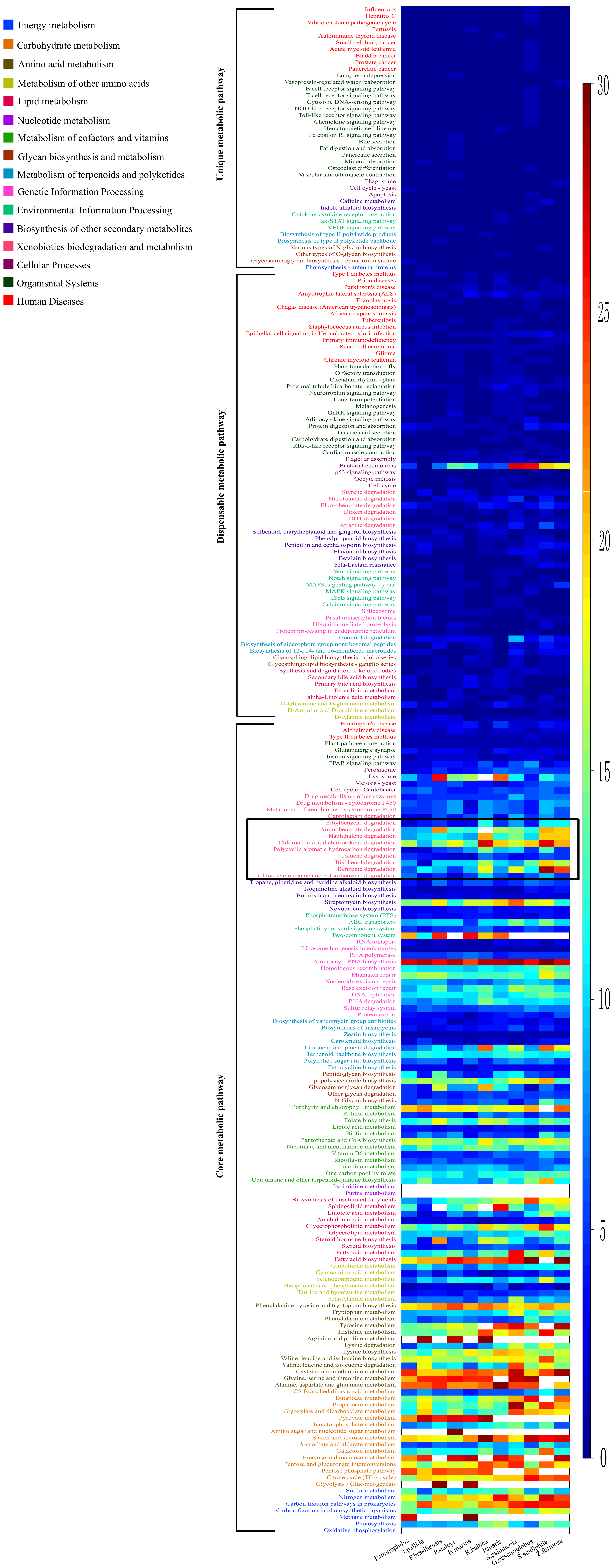

Supplement: Figure S3 — Metabolic analysis of the 11 Planctomycetaceae species. Color of the heatmap box means gene number, which indicate in the color bar at the right of the heatmap; the colored box in legend at the left shows the same meaning with the colored text in the heatmap. (PDF) [file pone.0086752.s003.pdf]
